# Decreased Lower Limb Phase Angle in Older People Is an Indicator of Standing and Gait Function, Regardless of Age

**DOI:** 10.3390/jcm14031023

**Published:** 2025-02-06

**Authors:** Daisuke Homma, Norio Imai, Dai Miyasaka, Moeko Yamato, Masafumi Ishisaki, Tsubasa Sugahara, Yoji Horigome, Hayato Suzuki, Yoichiro Dohmae, Naoto Endo, Izumi Minato, Hiroyuki Kawashima

**Affiliations:** 1 Division of Orthopaedic Surgery, Graduate School of Medical and Dental Sciences, Niigata University, 757, Asahimachi-dori Ichiban-cho, Chuo-ku, Niigata 951-8510, Japan; inskawa@med.niigata-u.ac.jp; 2 Department of Rehabilitation, Niigata Bandai Hospital, 2-2-8, Yachiyo, Chuo-ku, Niigata 950-0909, Japan; 3 Division of Comprehensive Musculoskeletal Medicine, Graduate School of Medical and Dental Sciences, Niigata University, 757, Asahimachi-dori Ichiban-cho, Chuo-ku, Niigata 951-8510, Japan; 4 Division of Orthopaedic Surgery, Niigata Bandai Hospital, 2-2-8, Yachiyo, Chuo-ku, Niigata 950-0909, Japan; 5 Division of Orthopaedic Surgery, Tachikawa General Hospital, 24-1, Asahioka, Nagaoka 940-8621, Japan; 6 Department of Orthopedic Surgery, Saiseikai Niigata Kenoh Hospital, 5001-1 Kamisugoro, Sanjo 955-0091, Japan; 7 Division of Orthopaedic Surgery, Niigata Rinko Hospital, 1-114-3 Momoyamacho, Higashi-ku, Niigata 950-0051, Japan

**Keywords:** phase angle, bioelectrical impedance analysis, healthy aging, muscle mass, motor function

## Abstract

**Background/Objectives**: The phase angle (PhA), as measured using bioelectrical impedance analysis (BIA), indicates muscle mass and quality. However, its relationship with age-related muscle changes and motor function and effective BIA assessment/intervention sites remains unclear. Herein, we evaluated age-related changes in PhA, explored the relationship between PhA and muscle mass, and identified effective sites for BIA. **Methods:** We included 131 healthy community-dwelling adults divided into older (≥65 years) and control (≤65 years) groups. PhA and muscle mass were measured using BIA with a device determining electrical resistance by transmitting a weak alternating current <90 μA. Motor function was measured using ground reaction force index, gait function, and grip strength. The relationships between muscle mass, PhA, and motor function were analyzed. **Results:** All values, excluding upper limb muscle mass, were lower in the older group, as were the rates of change in muscle mass and PhA in the lower limbs vs. the upper limbs. Lower limb PhA showed negative and positive correlations with the Timed Up and Go test and standing function, respectively; it was significantly associated with all motor functions. **Conclusions:** Regardless of age, muscle mass and PhA measured by BIA were high and low in the lower limbs, respectively, and lower limb PhA was related to walking and standing function. The decrease in lower limb muscle mass and PhA may accelerate with age. Given global population aging, easy-to-assess lower limb PhA linked to the movements necessary for independent living may be an effective site for assessment and intervention in clinical practice.

## 1. Introduction

The global trend of population aging presents a significant global health challenge [1]. Based on the proportion of individuals aged 65 and older, populations are categorized as aging (>7%), aged (>14%), super-aged (>21%), and ultra-aged (>28%) [2]. From 2000 to 2020, the global percentage of individuals aged ≥65 years increased from 6.9% to 9.3%, with future estimates indicating that this number will rise to 15.9% by 2050 and 22.4% by 2100 [3]. Consequently, fostering healthy aging has become a priority. According to the World Health Organization, healthy aging involves maintaining and developing functional abilities that contribute to well-being in older age [4,5]. Functional abilities stem from the interaction between intrinsic capacities and environmental factors [6]. These intrinsic capacities include physical abilities, highlighting the necessity of addressing the variability in health and functional status among older adults to promote healthy aging. As such, it is critical to establish strategies for the prevention, evaluation, and improvement of physical capacities.

Maintaining and improving basic movement abilities, including standing and walking, are essential for maintaining a healthy life and sociality and ensuring healthy aging. As muscles are required to perform these movements, objective evaluation, measurement, and collection of data related to muscle decline with age are important for maintaining and improving intrinsic abilities. While both muscle strength and mass decrease with age, the reduction in muscle strength is notably greater than the decrease in muscle mass [7,8]; therefore, muscle strength decline cannot be completely characterized by muscle mass decline alone [9]. The intercellular spaces in the skeletal muscle consist of muscle fibers (muscle cells), intramuscular extracellular fat, fibrous tissue, and extracellular fluid. Muscle atrophy is marked by both quantitative changes (such as a reduction in muscle cross-sectional area) and qualitative alterations (such as increases in fibrous tissue and intramuscular fat) [10]. Consequently, it is essential to assess both muscle quality and mass. Traditional methods for evaluating muscle include computed tomography (CT), magnetic resonance imaging (MRI), muscle biopsy, electromyography, and ultrasound; however, these methods are constrained by the requirement for large-scale equipment, skilled personnel, invasiveness, and the time needed for result interpretation and providing feedback to participants [11]. Recent research has revealed that vibratory arthrography can provide valuable information regarding the condition of joint structures; however, a standardized measurement method is lacking [12]. To overcome these difficulties, multi-frequency bioelectrical impedance analysis (BIA) has been applied. This method evaluates the phase angle (PhA), a marker of muscle mass and integrity, by transmitting a mild electrical current through the body. BIA offers a non-invasive, cost-effective, and portable solution, thereby enabling straightforward and quick assessments [11,13]. Consequently, the BIA method is easier and takes less time to perform than CT or MRI measurements and can be performed without staff having specialized skills or knowledge, making it an ideal measurement strategy in local and clinical settings.

Earlier studies on PhA using BIA have utilized whole-body values as indicators [14,15,16]; however, in recent years, studies have increasingly focused on values for each body part. A previous study comparing pre-frail adults (defined as the stage preceding frailty) with robust adults found that a significant decrease in PhA of the lower extremities was associated with motor function [17]. Furthermore, in older adults, the PhA of the lower limbs is significantly lower than that of the upper limbs and is linked to indicators of locomotive syndrome [18]. These reports indicate that muscle mass and PhA measurements using BIA for each part based on function may be more effective than whole-body measurements. Furthermore, previous studies have suggested that lower limb PhA measurements may effectively assess basic movements, including standing up and walking [17,18]. However, previous studies assessing the PhA of each body part in an older population did not include control participants [17,18]. The lack of a control group in previous studies is considered a limitation of this study. Furthermore, as there was no comparison between the elderly and control groups, this study did not clarify whether the relationship between the decline in PhA of the lower limbs and motor function was a characteristic of the elderly population. Furthermore, although there have been several studies that characterize diseases in the elderly population, there are none characterizing the PhA of each body part in younger individuals. Additionally, an effective evaluation site for BIA has not yet been identified in any age group. Using BIA to evaluate the differences and relationships between the muscle mass, PhA, and motor function of each body part in elderly and young groups will clarify whether the decline in lower limb PhA in elderly individuals is specific and will contribute to identifying an effective evaluation site for BIA. This characterization will also help to identify if muscle mass or PhA measured by BIA is a more effective indicator of motor function, including walking and standing, and will contribute to intervention considerations for improving motor function.

Expanding on prior research, we hypothesized that age-related muscle atrophy appears earlier in the lower limbs than in the upper limbs [19,20]. Additionally, muscle strength is observed to deteriorate at a rate three times greater than muscle atrophy, indicating the involvement of other factors in the observed reduction in muscle mass [21,22]. As such, effective efforts to maintain and improve lower limb muscle strength are likely to reduce the risk of falls in adults and contribute to maintaining an independent lifestyle. We believe that this is extremely important knowledge given the global context in which aging is a major social issue.

The aim of this study was to evaluate the differences in PhA and muscle mass at various body sites between older and younger groups and to thereby characterize the age-related changes in these values. Additionally, we sought to explore the relationship between PhA, muscle mass, and motor function related to standing and walking within each group and to identify effective points for evaluation and intervention.

## 2. Materials and Methods

### 2.1. Study Design and Evaluated Parameters

This research was conducted as a cross-sectional, analytical, and observational study. Physical function was assessed through upper and lower limb PhA and muscle mass, while motor function was evaluated using the timed up-and-go test (TUG), ground reaction force/weight during standing up (F/w), rate of force development/weight (RFD8.75/w), and grip strength. To assess the condition of the older group, the 25-Geriatric Locomotive Function Scale (GLFS-25) was applied to assess locomotive syndrome, while the Kihon Checklist (KCL) was used to evaluate frailty.

### 2.2. Participants

The study period was from 1 September 2022 to 31 March 2024. Volunteers living near Niigata and Shibata Cities in Niigata Prefecture, Japan, were recruited and underwent measurements at local facilities during the day. This study involved 134 healthy adult individuals. The inclusion criteria based on previous studies were as follows: (1) ability to walk independently without walking aids, (2) ability to live an independent daily life, and (3) being Japanese [18]. The exclusion criteria were as follows: (1) the presence of conditions that may affect daily life, including paralysis; (2) neurological diseases, including numbness; and (3) pacemaker use.

**Figure 1:**
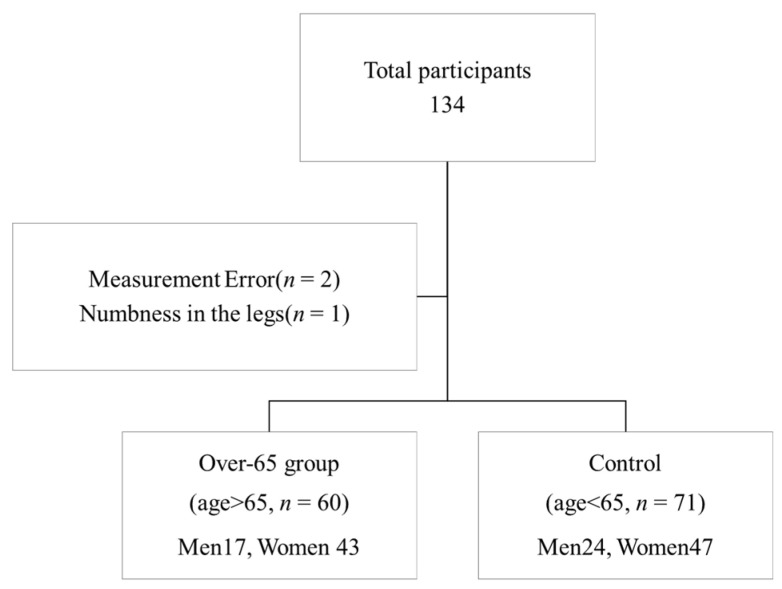
Patient recruitment flowchart. Overall, 134 cases were included during the measurement period; 3 participants (2 with measurement errors and 1 with numbness in the lower limbs) were excluded. Thus, the data analyzed were from 131 participants. Participants aged 65 years or older were placed in the older group (*n* = 60), while those aged 65 years or younger were assigned to the control group (*n* = 71).

Data were collected from 134 participants during the measurement period; 2 participants had measurement errors, and 1 participant had numbness in the lower limbs; therefore, 3 participants were excluded. Finally, the analysis included data from 131 participants (Figure 1). Per previous studies [23,24], participants aged ≥65 years were classified into the older group (*n* = 60), while those aged 65 years or younger were assigned to the control group (*n* = 71).

This research was conducted in line with the tenets of the Declaration of Helsinki and was approved by the Ethics Committee of Niigata Bandai Hospital (approval number: 2310-119; date: 18 October 2023). All participants provided written informed consent prior to participation.

### 2.3. PhA and Muscle Mass Measurements

**Figure 2:**
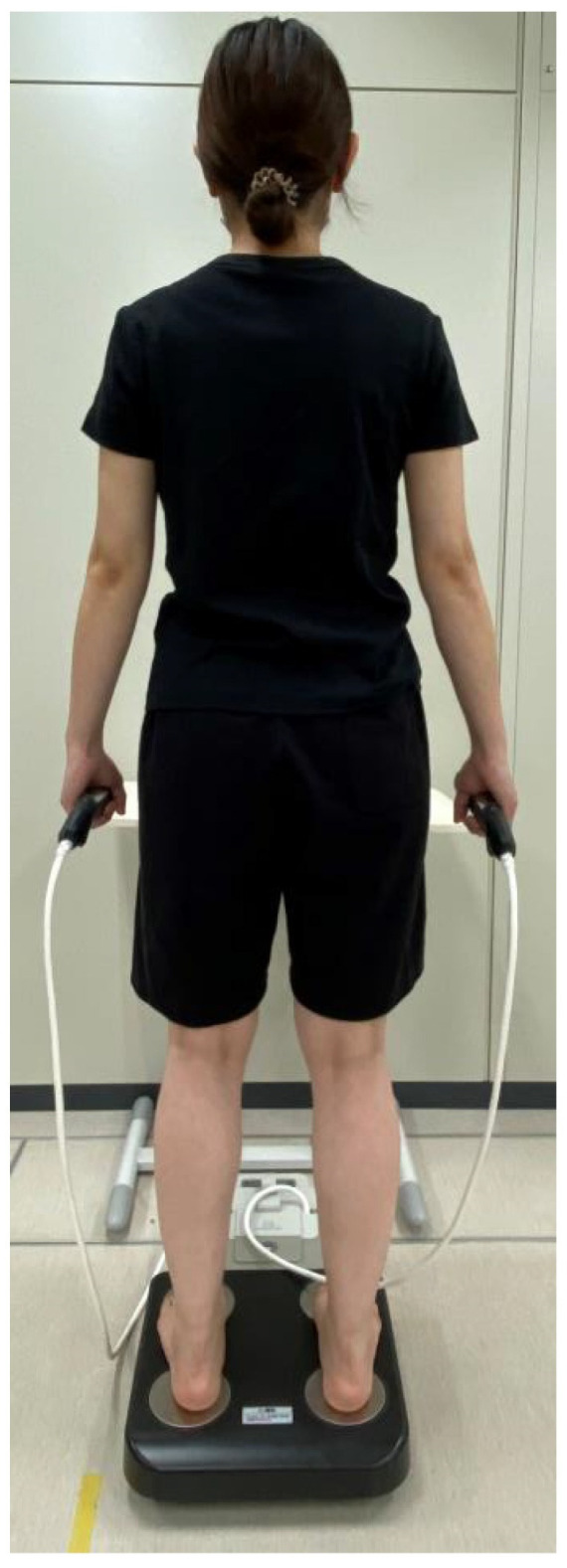
The BIA measurement position. Prior to taking measurements, the participants’ electrodes and skin were cleansed with alcohol. All measurements were conducted under uniform conditions. During the procedure, participants stood with bare feet placed on the toe and heel electrodes while holding the hand grip, with their arms hanging a few centimeters from their bodies.

All measurements were taken at a constant room temperature within the host facility. PhA and muscle mass were evaluated using a multi-frequency, 8-electrode body composition analyzer (MC-780A-N, Tanita, Tokyo, Japan). The devices and methods used for measurement have been employed in prior studies with comparable populations [17,18]. Prior to measurements, participants’ skin and electrodes were cleaned with alcohol, and all measurements were taken under consistent conditions. Participants stood while holding the hand grip, with their arms hanging several centimeters away from their bodies. The participants were barefoot during the measurements, with their heels and toes touching the electrodes (Figure 2). The BIA device utilized in this study assesses electrical resistance by transmitting a mild alternating current of less than 90 μA through the body. The measurement frequencies used were 5, 50, and 250 kHz, and the extracellular and intracellular water content of the participants’ bodies’ was measured directly. This BIA device uses an eight-electrode method and can measure the individual impedance of each body part; consequently, the muscle mass for the lower and upper limbs, as well as the total body, was computed separately. Muscle mass was normalized by dividing by body weight.

PhA was also evaluated using BIA as a marker of muscle quality. In this measurement, a weak alternating current of ≤90 µA was applied to the body to measure resistance (R) and reactance (Xc), with the measurement frequency set at 50 kHz. PhA was determined using the following formula: PhA (°) = [arc tangent (Xc/R) × (180/π)]. The PhA for the entire body was calculated using the value from the left side. For the upper and lower limbs, the PhA was determined by averaging the measurements from both sides.

### 2.4. TUG

The TUG test, used as an index in this study, was created by Podsiadlo and Richardson [25]. It is recognized for its high reliability [26] and shows strong correlations with the Berg Balance Scale (r = 0.81) and the Barthel Index for daily living activities (r = 0.78) [27]. Additionally, the TUG test has excellent inter-examiner reproducibility [28], which is why it was chosen as the walking function assessment in the present study. All participants were able to walk without the aid of a cane and were capable of performing activities of daily living independently. The chair used for the TUG test had no armrests and a seat height of 42 cm. Participants began the test seated in the chair. Upon receiving a signal to start, they were instructed to rise, walk around a pole 3 m away, and return to the chair, where the time to sit back down was also recorded. They were asked to walk briskly and complete the task to the best of their ability. All participants performed the TUG test without a cane, and the test was repeated twice, with the fastest completion time used as the representative value for each participant.

### 2.5. RFD8.75/w When Standing Up and F/w

**Figure 3:**
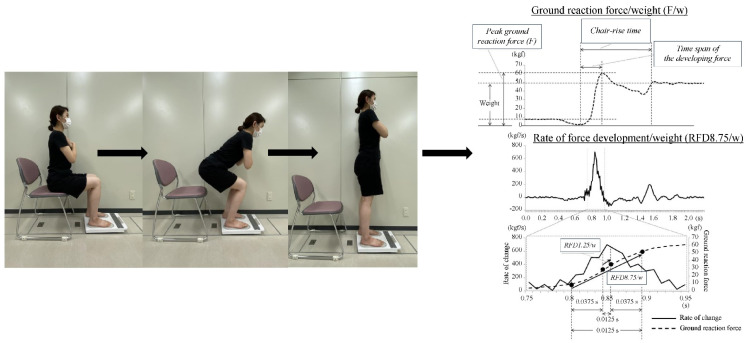
Measurement of ground reaction force index when standing up. The sit-to-stand movement was performed three times under maximum effort. The graphs show F/w and RFD8.75/w as indices representing the ground reaction force during standing. F/w is calculated as the maximum ground reaction force divided by body weight, while RFD8.75/w measures the rate of change in ground reaction force over 87.5 milliseconds, which includes 37.5 milliseconds prior to and 12.5 milliseconds following the maximum recorded increase, then converted to per second and normalized by body weight. This figure was derived from prior studies [30,31] with some modifications.

Motor function was assessed using a measurement device (Tanita, ZaRitz, Tokyo, Japan), which operates with a sampling rate of 80 Hz and a unit of 0.01 kgf/s·kg^−1^. During this task, participants were seated on a chair with a seat height of 42 cm, positioned for ease of standing up. The chair used was the same as that used for the TUG test. For the chair-sitting posture, the participants placed both feet 10 cm apart on the sensor, crossed their arms in front of their chest, and stood up three times with maximum effort. The chair rise test is a method with high inter-examiner reproducibility [29]. The ground reaction force coefficients, RFD8.75/w (kgf/s·kg^−1^) and F/w (kgf·kg^−1^), were measured during the task movements (Figure 3).

RFD8.75/w serves as an index for the rate of change during the peak increase in ground reaction force [32]. The rise in ground reaction force over 87.5 milliseconds, which includes 37.5 milliseconds before and 12.5 milliseconds after the maximum increase, was converted into per-second values and normalized by body weight. The highest measurement was taken after performing three trials.

F/w refers to the maximum vertical stepping force when standing up from a chair. It was calculated by dividing the peak ground reaction force by body weight [32]. From the three trials, the F/w corresponding to the highest RFD8.75/w, which measures the rate of change during the peak increase in ground reaction force, was selected. Because the ground reaction force index during these sit-to-stand movements is known to be an effective predictor of falls [32], this study measured it as an indicator of motor function.

### 2.6. Grip Strength

Grip strength was assessed as outlined in previous studies [18,33]. Grip strength was assessed using a grip strength dynamometer (T.K.K5101, TAKEI, Niigata, Japan). In brief, participants stood while holding a handgrip dynamometer to measure the strength of their dominant hand. The test was performed twice, and the highest value was recorded.

### 2.7. Questionnaire

The older group participants may have suffered from geriatric diseases, frailty, and locomotive syndrome; as such, the presence of these conditions was assessed using questionnaires. Frailty was evaluated using the KCL [34], which has a maximum score of 25 points. This tool assesses the extent of frailty, with higher scores indicating more severe frailty. Participants were classified into three groups based on their scores: frail (≥8 items applicable), pre-frail (4–7 items applicable), and robust (>3 items applicable).

The GLFS-25, a 25-item questionnaire developed by the Japanese Orthopaedic Association, was used to assess LS [35]. The higher the score on the GLFS-25, the higher the severity of the locomotive syndrome, with participants classified as robust (≤6 points), GLFS1 (7–16 points), GLFS2 (16–24 points), and GLFS3 (≥24 points).

Both the KCL and GLFS-25 were paper questionnaires and were assessed as continuous variables.

### 2.8. Statistical Analyses

Data analysis was carried out using SPSS version 29.0.1.0 (171) (IBM Corporation, Armonk, NY, USA). Data distribution was evaluated for normality using the Kolmogorov–Smirnov test. For variables with a normal distribution, values are presented as mean ± standard deviation, while non-normally distributed data are shown as median (interquartile range).

To evaluate the characteristics of the older group, comparisons of each measurement between the older and control groups were conducted. For normally distributed data, a two-sample *t*-test was applied, while the Mann–Whitney U test was applied for data that did not follow a normal distribution.

To explore the association between muscle mass, phase angle, and motor function for each body part within the groups, Pearson’s correlation coefficient was used for normally distributed variables, while Spearman’s rank correlation was applied to non-normally distributed data. A *p*-value of less than 0.05 was considered significant for all tests. Additionally, a post hoc analysis was performed to evaluate the statistical power (type II (β) error), with an effect size (d) of 0.5 and type I (α) error set at 0.05, based on the results of the two-sample *t*-test and Pearson correlation analysis. The two-sample *t*-test’s power value for the post hoc analysis was 0.808. Pearson’s correlation analysis revealed values of 0.987 and 0.995 for the older and control groups, respectively.

## 3. Results

### 3.1. Difference in Participant Characteristics Between the Two Groups

In this study, the participants were categorized into older (≥65 years old) and control (≤65 years of age) groups, comprising 60 (17 men, 43 women) and 71 (24 men, 47 women) participants, respectively. The participants in the older group could walk independently and live an independent life, with a KCL score of 3 (1–4) points and a 25-Geriatric Locomotive Function Scale score of 5 (1.75–11.25) points with no severe geriatric diseases progressing.

The older group had a mean age of 73.7 ± 4.5 years, height of 157.3 ± 7.0 cm, and weight of 54.9 (49.8–63.5) kg, while the control group had a mean age of 38 (27–48.7) years, height of 163.5 ± 8.7 cm, and weight of 55.8 (49.7–65.6) kg; thus, the participants in the older group were significantly older and shorter (Table 1).

Regarding muscle mass, the total body mass of the participants in the older group was 66.7 ± 7.0%, with an upper limb mass of 6.2 ± 0.8% and a lower limb mass of 23.9 ± 2.8%; while the control group had a total body mass of 70.7 ± 7.9%, an upper limb mass of 6.2 (5.6–7.6)%, and a lower limb mass of 26.9 ± 3.5%. Consequently, the older group exhibited lower total body and limb muscle mass compared to the control group.

The PhA for the older group was 5 (4.7–5.5)°, 5.1 (4.8–5.6)°, and 4.2 ± 0.6°, for the whole body, upper limbs, and lower limbs, respectively. In comparison, the PhA for the control group was 5.3 (4.8–5.8)° for the whole body, 5.7 ± 0.6° for the upper limbs, and 4.9 (4.4–5.6)° for the lower limbs. Therefore, all PhA values in the older group were lower than those in the control group (Table 1).

The ratio of lower limb muscle mass and PhA to upper limb values (Table 1) was also significantly lower in the older group (muscle mass, 384.2 ± 28.1%; PhA, 80.6 ± 8.1%) compared to the control group (muscle mass, 414.8 ± 36.3%; PhA, 88.7 ± 10.0%). Additionally, we examined the differences in muscle mass and PhA between the upper and lower limbs within each group. Overall, we found that muscle mass was significantly higher in the lower limbs, while PhA was significantly higher in the upper limbs for both groups (Table 2). As such, lower limb muscle mass exceeded that of the upper limbs, irrespective of age, while upper limb PhA was greater than lower limb PhA. Moreover, muscle mass and PhA declined with age, except for upper limb muscle mass; however, the ratio of lower limb muscle mass and PhA to upper limb values was smaller in the older group, indicating that aging may specifically affect the reduction in lower limb muscle mass and PhA.

### 3.2. Relationship Between Muscle Mass, PhA, and Exercise Function

Within the older group, the only item significantly correlated with all motor functions measured in this study was the lower limb PhA. The correlation coefficients between lower limb PhA and all motor functions were 0.445, 0.300, −0.277, and 0.493 for F/w, RFD/w, TUG, and grip strength, respectively.

In the control group, total body muscle mass relative to body weight, lower limb muscle mass relative to body weight, and PhA were all significantly correlated with all motor functions. Among these, the lower limb PhA had a stronger correlation with F/w (*r* = 0.693), RFD/w (*r* = 0.718), TUG (*r* = −0.563), and grip strength (*r* = 0.705) than the other body composition parameters (Table 3).

## 4. Discussion

### 4.1. Key Results

Overall, this study showed that in both the older (aged ≥ 65 years) and control groups, muscle mass was greater in the lower limbs, while the PhA was lower in the lower limbs, with both groups exhibiting similar patterns. However, the ratio of lower limb muscle mass and PhA compared to the upper limbs was notably reduced in the older group. Furthermore, the reduction in PhA, which reflects muscle mass and quality in the lower limbs, increased with age. In both groups, the lower limb PhA was associated with all motor functions in the older group. In the control group, total body muscle mass, lower limb muscle mass, and PhA for each body segment were all connected to motor functions, with the strongest correlation observed for lower limb PhA. Consequently, lower limb PhA may prove to be a useful index for assessing muscle mass and PhA via bioelectrical impedance analysis (BIA), regardless of age, due to its connection with motor functions related to standing and walking. This research is the first to compare older and control groups to evaluate differences in muscle mass and PhA at specific body sites using BIA. Moreover, it examined the relationship between motor functions related to walking and standing and identified key BIA assessment sites, an area not previously explored. Overall, these findings provide new insights for designing strategies to prevent and enhance muscle mass and quality for healthy aging.

### 4.2. Muscle Mass, PhA, and Motor Function Characteristics Assessed by BIA in Both Groups

The participants enrolled in this study needed to meet several criteria, including the ability to walk independently and live an independent daily life. Furthermore, as geriatric diseases can affect the results with age, frailty, and locomotive syndrome indices were evaluated, and the older group had robust participants based on the KCL and GLFS-25 results [34,35].

A comparison of the older and control groups revealed that muscle mass and PhA in the older group were significantly decreased, except for upper limb muscle mass. Furthermore, the motor function items associated with standing up and walking, except for grip strength, were also significantly decreased in the older group. PhA serves as an indicator influenced by muscle mass, meaning the reduction in both muscle mass and PhA in the older group for all body parts, except for upper limb muscle mass, is understandable, given that lower limb atrophy typically occurs before upper limb atrophy with advancing age [19,20]. In the present study, there was no observed difference in upper limb muscle mass between the two groups; however, a notable difference is anticipated as the older group continues to age.

The purpose of this study was to examine age-associated alterations in muscle mass and PhA. Overall, our results revealed that both groups displayed similar patterns, with lower limb muscle mass being larger than upper limb muscle mass and lower limb PhA being smaller than that of the upper limbs. However, the changes in lower limb muscle mass and PhA, compared to the upper limbs, were significantly less pronounced in the older group than the control group. Consequently, muscle mass and PhA in the lower limbs decline with age.

### 4.3. Connection Between Muscle Mass, PhA, and Motor Performance as Assessed by BIA

The relationship between muscle mass, PhA, and motor function was assessed in each group using BIA. In the older group, all motor functions were linked exclusively to lower limb PhA. In the control group, all factors, apart from upper limb muscle mass, showed significant associations with motor function, with the strongest correlation observed between motor function and lower limb PhA. Consequently, the lower limb PhA evaluation using the BIA may be effective for assessing motor function in both the older and control groups.

Age-related alterations in muscle include both muscle mass and strength changes. Muscle mass remains relatively stable from 18 to 60 years but begins to decline after the age of 60 [36]. Furthermore, neural factors associated with movement decrease [37,38], including alterations in command drive [39,40], spinal reflex excitability [41,42], and motor unit discharge rate [43]. As the older group in this study was ≥65 years of age, nervous system factors may have also been affected. Changes in the nervous system due to aging can influence exercise performance and may be linked to factors beyond just muscle mass and PhA. We believe that a few parameters correlated with muscle mass and PhA of each body part measured by BIA. However, lower limb PhA showed associations with all the variables evaluated in this study. We attribute this to the specific characteristics of PhA as well as the components of the measurements. In this study, a comparison of the two groups showed that both lower limb muscle mass and PhA declined with age. Additionally, PhA proved to be a more responsive indicator of motor function than muscle mass [17,18]. Moreover, the evaluation indices for motor function measured in this study were mostly related to standing up and walking; therefore, we believe only the lower limb PhA was involved. Lower limb PhA, which is also associated with grip strength and quadriceps strength [44], likely reflects the muscle strength of the entire body. Therefore, as in a prior study [17], we considered the correlation between grip strength and lower limb PhA in the present study. The association between lower limb PhA with floor reaction force and TUG during standing revealed in this study is supported by those of prior studies investigating the relationship between PhA and motor function in pre-frail participants and older adults [17]. As the participants in this study were robust, lower limb PhA may be an effective evaluation index for the motor functions necessary for independent living, including standing and walking, regardless of the presence or absence of geriatric diseases, such as pre-frailty.

When compared to the older group, the control group displayed significant associations with all motor functions, excluding upper limb muscle mass, with the most pronounced correlation observed in lower limb PhA. Given that the control group participants were younger, age-related changes in the nervous system were not a factor. Furthermore, body structure is thought to directly influence motor function, and we observed notable correlations with motor performance across various measures. In the older group, PhA showed a stronger association with motor function than muscle mass, and a similar pattern was observed in the control group, suggesting that PhA may be a more sensitive indicator than muscle mass for assessing and improving motor function, especially in the lower limbs.

### 4.4. Clinical Applications

Overall, the results of this study suggest that the decrease in muscle mass and PhA of the lower limbs accelerates with age, while lower limb PhA may be an effective evaluation index, regardless of age, owing to its correlation with motor function. Prior studies have thus far failed to report an effective measurement site for evaluating indices using BIA. The results of the present study indicate that lower limb PhA changes, rather than lower limb muscle mass, should be assessed as an effective evaluation metric for BIA in clinical practice, while interventions to combat age-related muscle degeneration and to maintain standing up and walking function should be focused accordingly. Therefore, we recommend lower limb PhA measurement as a method of assessing important factors for independent living, including walking and standing up, regardless of age. In the future, the practicality of this study is expected to be greatly improved by clarifying the cutoff value for PhA in the relationship between lower limb PhA and motor function.

As people age, they become increasingly frail and require continued care, making early prevention of frailty necessary. However, one study comparing pre-frail individuals with a non-frail control group revealed no difference in lower limb muscle mass, although lower limb PhA was shown to decline [18]. Therefore, assessment and intervention focusing on lower limb PhA may be applicable not only to healthy older adults but also to frail and pre-frail individuals and to maintaining lower limb strength, gait, and function during the aging process.

The present study clarified the effectiveness of lower limb PhA, but as a specific measure, several methods have been proposed to improve muscle quality, including protein intake [45], aerobic exercise [46], and resistance training [47]. These are common methods for managing general muscle hypertrophy; however, we believe that they can also improve muscle quality. In the future, it is expected that specific methods for achieving healthy aging that could be applied to a variety of people will be proposed by characterizing the effects of detailed intervention methods, such as muscle contraction patterns, exercise time, and the effectiveness of exercise under low load. These interventions may contribute to the prevention of muscle deterioration and improvement of muscle function.

### 4.5. Limitations

This study had several limitations linked to participant recruitment and the local measurement of parameters. First, we measured muscle mass and PhA using the BIA method, but because the measurement models are linked, we believe that the effectiveness of PhA and muscle mass can be better examined by measuring muscle mass using ultrasound or MRI. Further, the sample size was small, and the data were analyzed in a mixed-sex group. As muscle mass and PhA measured by BIA are values that are affected by gender, analysis and comparison by gender are necessary; however, we had difficulty gathering a sufficient number of subjects to conduct analyses stratified by gender. In the future, more detailed analysis will be possible by conducting analysis by gender. In addition, the elderly people aged 65 years or older who were subjects of this study were evaluated for locomotive syndrome and frailty indicators, and although they were able to live independently, their physical activity levels, comorbid diseases, and nutritional status were not assessed in detail. These measurement items may provide insight into health status and PhA, so urgent investigation is required. Furthermore, as BIA is affected by the body’s water content, a detailed evaluation by adjusting the water and food intake and estimating the amount of excretion before measurement could be conducted. However, as the participants of this study were people living in the local area and the measurements were conducted in a local area rather than in a laboratory, adjusting the amount of water intake, excretion, and food ingested before the measurement was difficult. When more detailed surveys are conducted in the future, more precise measurements will be possible through the application of more detailed regulations, such as controlling the amount of food and fluid intake and excretion and the timing of measurement after intake. By standardizing the timing of measurements, more detailed measurements using BIA will be possible. Moreover, obtaining detailed background information, including the degree of edema and history of heart disease, was difficult. These factors may influence the results measured by BIA and need to be considered in future studies. Furthermore, the participants of this study met multiple criteria and could live independently. Therefore, generalizing these results to patients with illnesses may be difficult. Finally, as a cross-sectional observational study, this study was unable to clarify age-related changes in muscle mass, PhA, or motor function. Therefore, we believe that longitudinal research should be conducted in the future to elucidate age-related changes. Although these limitations may affect the relationship between PhA and measured motor function, the finding that lower limb PhA is strongly related to locomotion supports previous research [17]. Considering the physiological changes associated with aging, the decrease in PhA, which reflects the physiological health of muscle quality and cells, may warrant consideration, particularly given the pronounced lower limb PhA decrease noted in the older group. Further investigation is needed to explore the changes. Additionally, caution should be exercised in generalizing the findings due to multiple study limitations.

## 5. Conclusions

Overall, this study found that muscle mass measured using the BIA was high in the lower limbs, regardless of age, while PhA was low in the lower limbs. Aging may accelerate the decline in lower limb muscle mass and PhA. Furthermore, lower limb PhA was associated with functions, including standing up and walking in both groups. The correlation coefficient was the highest in the control group (aged ≤65 years), suggesting that lower limb PhA may be an effective site for assessment and intervention using BIA for prevention of muscle decline. While this study had limitations, such as not taking the subjects’ personal factors or gender into account, large-scale measurements are necessary in the future. Nevertheless, these results clearly show that lower limb PhA is related to the motor functions essential for daily life, regardless of age. Because lower limb PhA can be easily measured using BIA, it is expected that incorporating the evaluation of lower limb PhA into daily clinical care will contribute to the maintenance and improvement of independent lifestyle habits.

## Figures and Tables

**Table 1:** Differences in demographic information between the older and control groups.

	Older Group	Control	*p*-Value
Age	73.7 ± 4.5	38 (27–48.7)	<0.01
Height (cm)	157.3 ± 7.0	163.5 ± 8.7	<0.01 †
Weight (kg)	54.9 (49.8–63.5)	55.8 (49.7–65.6)	0.567
Whole-body muscle mass/weight (%)	66.7 ± 7.0	70.7 ± 7.9	<0.01 †
Upper body muscle mass/weight (%)	6.2 ± 0.8	6.2 (5.6–7.6)	0.116
Lower body muscle mass/weight (%)	23.9 ± 2.8	26.9 ± 3.5	<0.01 †
Whole-body phase angle (°)	5 (4.7–5.5)	5.3 (4.8–5.8)	0.045
Upper phase angle (°)	5.1 (4.8–5.6)	5.7 ± 0.6	<0.01
Lower phase angle (°)	4.2 ± 0.6	4.9 (4.4–5.6)	<0.01
Lower muscle mass/upper muscle mass (%)	384.2 ± 28.1	414.8 ± 36.3	<0.01 †
Lower phase angle/Upper phase angle (%)	80.6 ± 8.1	88.7 ± 10.0	<0.01 †
F/w (kgf·kg^−1^)	1.3 ± 0.09	1.4 (1.3–1.5)	<0.01
RFD8.75/w (kgf·kg^−1^)	10.9 ± 1.7	13.2 ± 2.6	<0.01 †
TUG (s)	6.1 (5.5–6.7)	4.8 ± 0.7	<0.01
Grip strength (kg)	25.9 (23.7–30.0)	27.5 (23.8–37.3)	0.121
Kihon Checklist score (points)	3 (1–4)	-	
25-Geriatric Locomotive Function Scale score (points)	5 (1.75–11.25)	-	

F/w, force/weight when standing up; RFD8.75/w, rate of force development/weight; TUG, timed up-and-go. Normally distributed data: mean ± standard deviation; non-normally distributed data: Median value (interquartile range); †: Paired *t*-test; no mark: Wilcoxon signed rank test. The significance level for each examination was set at a two-tailed *p*-value of <0.05.

**Table 2:** Comparison of muscle mass and phase angle between the upper and lower limbs in the older and control groups.

Older Group	Upper Limb	Lower Limb	*p*-Value
Mass (%)	6.2 ± 0.8	23.9 ± 2.8	*p* < 0.01 †
Phase angle (°)	5.1 (4.8–5.6)	4.2 ± 0.6	*p* < 0.01
Control	Upper	Lower	*p*-Value
Mass (%)	6.2 (5.6–7.6)	26.9 ± 3.5	*p* < 0.01
Phase angle (°)	5.7 ± 0.6	4.9 (4.4–5.6)	*p* < 0.01

†: Paired *t*-test; no mark: Wilcoxon signed rank test. The significance level for each examination was set at a two-tailed *p*-value of <0.05. Normally distributed data are presented as mean ± SD, and non-normally distributed data are presented as median (IQR).

**Table 3:** Relationship between muscle mass, PhA, and motor function between the older and control groups.

Older Group		F/w	RFD/w	TUG	Grip Strength
Whole-body muscle mass/weight	*r*	0.293	0.151	−0.113	0.249
	*p*	0.023 †	0.247 †	0.388	0.054
Upper muscle mass/weight	*r*	0.313	0.109	−0.139	0.364
	*p*	0.014 †	0.405 †	0.287	*p* < 0.01
Lower muscle mass/weight	*r*	0.328	0.211	−0.200	0.262
	*p*	0.010 †	0.104 †	0.124	0.043
Whole-body phase angle	*r*	0.232	0.041	−0.010	0.618
	*p*	0.073	0.753	0.936	*p* < 0.01
Upper phase angle	*r*	0.271	0.098	−0.053	0.672
	*p*	0.035	0.454	0.684	*p* < 0.01
Lower phase angle	*r*	0.445	0.300	−0.277	0.493
	*p*	*p* < 0.01 †	*p* < 0.01 †	0.031	*p* < 0.01
Control		F/w	RFD/w	TUG	Grip Strength
Whole-body muscle mass/weight	*r*	0.654	0.542	−0.370	0.440
	*p*	*p* < 0.01 †	*p* < 0.01 †	*p* < 0.01 †	*p* < 0.01
Upper muscle mass/weight	*r*	0.592	0.452	−0.177	0.512
	*p*	*p* < 0.01	*p* < 0.01	0.139	*p* < 0.01
Lower muscle mass/weight	*r*	0.650	0.553	−0.412	0.437
	*p*	*p* < 0.01 †	*p* < 0.01 †	*p* < 0.01 †	*p* < 0.01
Whole-body phase angle	*r*	0.631	0.591	−0.354	0.665
	*p*	*p* < 0.01	*p* < 0.01	*p* < 0.01	*p* < 0.01
Upper phase angle	*r*	0.643	0.585	−0.325	0.648
	*p*	*p* < 0.01 †	*p* < 0.01 †	*p* < 0.01 †	*p* < 0.01
Lower phase angle	*r*	0.693	0.718	−0.563	0.705
	*p*	*p* < 0.01	*p* < 0.01	*p* < 0.01	*p* < 0.01

F/w, force/weight when standing up; RFD8.75/w, rate of force development/weight; TUG, timed up-and-go. Normally distributed data: mean ± standard deviation; non-normally distributed data: Median value (interquartile range). †: Pearson; no mark: Spearman. The significance level for each examination was set at a two-tailed *p*-value of <0.05.

## Data Availability

The data presented in this study are available on request from the corresponding author.

## Abbreviations

The following abbreviations are used in this manuscript:

PhA	phase angle: An indicator reflecting the physiological function of cells believed to reflect muscle quality.
BIA	bioelectrical impedance analysis: A method for assessing muscle mass and quality
TUG	Timed Up and Go test: Gait function index
F/w	ground reaction force/weight: A ground reaction force index when standing up the reflecting the force exerted.
RFD8.75/w	rate of force development 8.75/weight: A floor reaction force index when standing up and reflects the rate of muscle exertion.
KCL	Kihon Checklist: Frailty assessment index
GLFS-25	25-Geriatric Locomotive Function Scale: Evaluation index of locomotive syndrome
